# Platelets, Constant and Cooperative Companions of Sessile and Disseminating Tumor Cells, Crucially Contribute to the Tumor Microenvironment

**DOI:** 10.3389/fcell.2021.674553

**Published:** 2021-04-16

**Authors:** Wolfgang M. J. Obermann, Katrin Brockhaus, Johannes A. Eble

**Affiliations:** Institute of Physiological Chemistry and Pathobiochemistry, University of Münster, Münster, Germany

**Keywords:** platelets, tumor microenvironment, coagulation, thrombus formation, fibrin, tissue factor, CLEC-2, integrins

## Abstract

Although platelets and the coagulation factors are components of the blood system, they become part of and contribute to the tumor microenvironment (TME) not only within a solid tumor mass, but also within a hematogenous micrometastasis on its way through the blood stream to the metastatic niche. The latter basically consists of blood-borne cancer cells which are in close association with platelets. At the site of the primary tumor, the blood components reach the TME via leaky blood vessels, whose permeability is increased by tumor-secreted growth factors, by incomplete angiogenic sprouts or by vasculogenic mimicry (VM) vessels. As a consequence, platelets reach the primary tumor via several cell adhesion molecules (CAMs). Moreover, clotting factor VII from the blood associates with tissue factor (TF) that is abundantly expressed on cancer cells. This extrinsic tenase complex turns on the coagulation cascade, which encompasses the activation of thrombin and conversion of soluble fibrinogen into insoluble fibrin. The presence of platelets and their release of growth factors, as well as fibrin deposition changes the TME of a solid tumor mass substantially, thereby promoting tumor progression. Disseminating cancer cells that circulate in the blood stream also recruit platelets, primarily by direct cell-cell interactions via different receptor-counterreceptor pairs and indirectly by fibrin, which bridges the two cell types via different integrin receptors. These tumor cell-platelet aggregates are hematogenous micrometastases, in which platelets and fibrin constitute a particular TME in favor of the cancer cells. Even at the distant site of settlement, the accompanying platelets help the tumor cell to attach and to grow into metastases. Understanding the close liaison of cancer cells with platelets and coagulation factors that change the TME during tumor progression and spreading will help to curb different steps of the metastatic cascade and may help to reduce tumor-induced thrombosis.

## Introduction

It may not be obvious to consider platelets and coagulation factors as components of the tumor microenvironment (TME), as they are blood components while the TME of a solid tumor mass consists of cancer cells, resident and infiltrating cells, and the dense network of extracellular matrix (ECM) (Iozzo and Sanderson, 2011; Theocharis et al., 2016; Henke et al., 2019; Martins Cavaco et al., 2020). Resident stroma cells may undergo a cancer cell-induced differentiation into cancer-associated fibroblasts (CAFs) (Pietras and Ostman, 2010; Kalluri, 2016; Wang et al., 2017), which remodel the ECM in a tumor-supportive manner (Eble and Niland, 2019). Also, infiltrating immune cells are influenced by tumor cells and thus attenuate their tumor-suppressive properties (Huber et al., 2020; Sadeghalvad et al., 2020; Yang et al., 2020). The non-cellular ECM, mostly synthesized, secreted, and arranged by CAFs, consists of collagen-containing fibrils, water-resorbing proteoglycans and glycosaminoglycans and a plethora of other ECM-proteins (Eble and Niland, 2019; Niland and Eble, 2020). Together, they form the scaffold, which keeps the tissue in shape and provides a stiff collagenous capsule of desmoplastic tumors (Leight et al., 2017; Mohammadi and Sahai, 2018; Martins Cavaco et al., 2020). Moreover, the ECM scaffold tethers growth factors, forming the morphogenic stage for cell differentiation (Lodyga and Hinz, 2020; Wei et al., 2020). Also the presence of metabolic components, such as protons, lactate, reactive oxygen species (ROS), contribute to the typical environment of cancer cells in a solid tumor mass (Oudin and Weaver, 2016).

Primarily, the site of neoplasia within epithelia or stroma tissue is not connected directly to the blood. The tumor mass seems far away from the blood, the usual environment of platelets and coagulation factors. However, in at least two scenarios, cancer cells and the blood components come close to each other: either, when the hypoxic TME induces angiogenic ingrowth of sprouting capillaries into the tumor mass (De Palma et al., 2017; Klein, 2018; Zanotelli and Reinhart-King, 2018), or when cancer cells hematogenously disseminate from their prime tumor site to colonize distant organs (Erpenbeck and Schon, 2010; Schlesinger, 2018). In both cases, cancer cells come in close contact with the blood components. Consequentially, platelets and coagulation factors play a crucial role in tumor progression and metastasis (Menter et al., 2017; Burbury and MacManus, 2018; Mege et al., 2019).

The liaison between tumor cells and platelets is clinically relevant, as tumor-induced thromboembolism is the second leading cause of death in cancer patients (Gay and Felding-Habermann, 2011a; Connolly and Francis, 2013; Mege et al., 2019; Palacios-Acedo et al., 2019). This frequent complication of enhanced and inappropriate platelet activation and blood coagulation in cancer patients has been known for one and a half century, since the physician Armand Trousseau described this type of thrombophlebitis in 1865 (Trousseau, 1865), later generally named Trousseau syndrome (Han et al., 2014).

This review highlights the molecular interactions of platelets and the coagulation system with tumor cells, thereby showing that these blood components are constituents of the TME, even only temporarily, but with an important impact on tumor progression and hematogenous metastasis.

## Platelets, the Cellular Players in Hemostasis

Platelets are anuclear cells with a diameter of 2–5 μm and a thickness of about 0.5 μm, which float in the blood stream at a density of 150,000–400,000 per μl. After they pinch off from the large megakaryocytes in the bone marrow, these small cells spend their lifetime of around 5–7 days in the blood circulation, unless they detect a damage within the endothelium-lined blood vessel wall (Andrews and Berndt, 2004). They are also recruited to more severe tissue damages, where both the endothelial lining and the underlaying basement membrane are breached, and the adventitial stroma tissue around the blood vessel is injured. There, platelets attach and close the wound. Thus, they stop the blood leakage into the tissue and fulfill their hemostatic functions, for which they are predominantly known (Andrews and Berndt, 2004, 2008; Gale, 2011; Broos et al., 2012; Menter et al., 2017).

Attachment is mediated by several cell adhesion molecules (CAMs), which not only anchor the platelets to the wound area, but also trigger signals within the platelets, that activate them (Broos et al., 2012). Activation of platelets is accompanied by a remarkable shape change (Yeung et al., 2018). Quiescent platelets have a discoid shape, which upon activation changes into a stellate-like appearance with pronounced platelet spreading and formation of lamellipodia and numerous filipodia. These protrusions enable platelets to entangle with each other, to spread on an adhesive substrate, generally at the injury site of a wounded blood vessel, and to form the primary thrombus (Gale, 2011; Broos et al., 2012; Berndt et al., 2014). Another step of platelet activation is degranulation. Platelets contain different types of granules, among them α-granules and dense granules, which upon platelet activation undergo exocytosis and release their contents (Gay and Felding-Habermann, 2011a). The former contain (i) ECM-proteins, such as von-Willebrand factor (vWF), fibrinogen, thrombospondin, (ii) coagulation factor V and VIII, the latter associated with vWF, (iii) growth factors, such as platelet-derived growth factor (PDGF), and (iv) membrane-anchored CAMs, such as P-selectin, which thus becomes exposed on the platelet surface exclusively after platelet activation. Dense granules are rich in adenosine diphosphate (ADP), adenosine triphosphate (ATP), serotonin, and histamine. Whereas the release of the ECM proteins, especially vWF, increases the adhesion of platelets (Grassle et al., 2014; Lancellotti et al., 2019), the purine nucleotides attract and activate additional platelets thereby increasing the size of the primary thrombus (Jamasbi et al., 2017; Yeung et al., 2018).

Platelet adhesion is crucial for thrombus formation (Andrews and Berndt, 2008; Broos et al., 2012; Jamasbi et al., 2017; Huang et al., 2019). Several CAMs enable platelets to attach firmly to an injured blood vessel wall, which is necessary to withstand the shear forces of the blood stream (Kunicki, 2001; Andrews and Berndt, 2008; Hansen et al., 2018). In addition to their mechanical functions, most of the CAMs also fulfill signaling functions, via which platelets perceive the environmental cues of adhesion (Yeung et al., 2018). Thus, adhesion triggers platelet activation, which in turn reinforces the binding activity of platelet CAMs to their ECM ligands. Amongst the plethora of platelet CAMs, we will focus on the vWF-binding GPIb-complex, on the collagen-binding receptors, glycoprotein (GP) VI and integrin α2β1, and on the fibrin receptor, integrin αIIbβ3 (Kunicki, 2001; Gay and Felding-Habermann, 2011a; Jamasbi et al., 2017; Martins Lima et al., 2019).

The vWF-receptor consists of four transmembrane protein chains, GPIbα, GPIbβ, GPIX, GPV, which assemble into the GPIb-complex in a 2:2:2:1 stochiometry within the platelet membrane (Kunicki, 2001; Andrews et al., 2003; Quach and Li, 2020). The GPIbα chain harbors the binding site for vWF (Lof et al., 2018; Lancellotti et al., 2019). vWF is a large ECM molecule consisting of several modules, which are assigned to four different types of domains; A, B, C, and D (Lancellotti et al., 2019). It is stored in the Weibel-Palade bodies of endothelial cells and after exocytosis multimerizes into a scaffold within the subendothelial basement membrane. In solution, it takes on a globular structure, in which also domain A1 with its GPIb-binding site is in a cryptic state (Huck et al., 2014; Lof et al., 2018). Upon association with other immobilized ECM proteins, e.g., with collagens via its A3 domain, and under hydrodynamic shear forces, vWF unwinds into an extended shape, which makes its A1 domain accessible to the platelet GPIb complex (Huck et al., 2014; Hansen et al., 2018). Thus, vWF bridges platelet attachment to collagen even under high blood flow rates, such as observed within the arterial branch of the circulatory system (Kulkarni et al., 2000; Hansen et al., 2018). Moreover, the GPIb-complex-mediated attachment of platelets to vWF and collagen under high shear forces results in stretching of the GPIb-chain and thus triggers an activating signal which is further transduced via the phosphoprotein-binding adaptor protein 14-3-3 within the platelet (Chen et al., 2018; Scharf, 2018b; Quach and Li, 2020).

The direct interaction of platelets with collagens is mediated via two distinct receptors, GPVI and α2β1 integrin (Nuyttens et al., 2011). Apparently, GPVI is physically associated in the platelet membrane with the GPIb complex (Arthur et al., 2005). GPVI is a type I transmembrane protein consisting of an extracellular tandem domain of two immunoglobulin C2 (Ig C2)-like modules, a glycosylated mucin-like stalk domain, a transmembrane and short cytoplasmic domains (Nieswandt and Watson, 2003). The Ig C2-like domains recognize bundles of collagen molecules, which contain the posttranslationally modified hydroxyproline residues that are essential for binding (Knight et al., 1999). Binding to collagen depends on the N-linked glycoconjugates of GPVI (Kunicki et al., 2005). High affinity binding of collagen also requires dimerization of GPVI receptors, which then signals via the associated Fc-receptor γ-chain (FcRγ) (Miura et al., 2002; Nieswandt and Watson, 2003). Deficiency of GPVI (Kato et al., 2003) or its inhibition by antibodies or pharmaceuticals (Nieswandt et al., 2001; Martins Lima et al., 2019) affect thrombus formation, although hemostasis, measured as bleeding time, is affected only mildly. In contrast to GPVI, the other collagen receptor on platelets, α2β1 integrin, belongs to the large family of integrins, consisting of two non-covalently associated subunits, α and β (Madamanchi et al., 2014; Zheng and Leftheris, 2020). The two integrin chains jointly form a globular head domain, which harbors the ligand binding site (Arnaout et al., 2005). All collagen-binding integrins contain an additional A-domain, which sits on top of the α-subunit propeller domain and is responsible for collagen binding (Emsley et al., 2000; Eble, 2005). The collagen ligands do not necessarily need to bear hydroxyproline residues (Perret et al., 2003; Niland et al., 2011), but the triple helical array of a specific collagenous integrin binding motif is indispensably required (Kühn and Eble, 1994; Hamaia and Farndale, 2014). Integrins undergo substantial changes in their conformation, which is influenced by ligand occupancy and, in turn, influences ligand binding (Arnaout et al., 2005; Luo et al., 2007; Hanein and Volkmann, 2018). Moreover, upon collagen binding, α2β1 integrins cluster on the platelet surface, likely mirroring the supramolecular array of integrin binding sites on collagen fibrils (Lima et al., 2018). This is similar to the ligand-induced clustering of GPVI and other CAMs on platelets (Ozaki et al., 2013; Poulter et al., 2017), which triggers a signaling cascade and thus induces platelet activation and aggregation (Humphries et al., 2019). The redundancy of the collagen receptors, GPVI and α2β1 integrin, on platelets has prompted several discourses and models, as the individual deletion of either of them did not entirely abolish collagen-induced platelet activation (He et al., 2003; Marjoram et al., 2014). Their interplay in collagen-induced platelet activation is still not entirely clear. Apparently, the two receptors influence each other in their signaling potential, as activation of GPVI seems to be regulated by α2β1 integrin (Atkinson et al., 2003), and *vice versa* (Inoue et al., 2003). The signals of both receptors convene into an integrating network (Lima et al., 2018; Izquierdo et al., 2020). Yet, another model postulates that GPVI is more involved in signal transduction, whereas α2β1 integrin firmly attaches the platelet mechanically to the collagen thereby allowing the forming thrombus to withstand the shear forces of the blood (Kunicki, 2001; Jarvis et al., 2004; Pugh et al., 2010).

The most numerous adhesion receptor with 50,000–100,000 molecules on the surface of a quiescent platelet and with many more molecules exposed upon platelet activation is the fibrin receptor, integrin αIIbβ3 (Coller, 2015; Huang et al., 2019). Not only the numbers of surface-exposed integrin αIIbβ3 molecules, but also the activation state of these fibrin receptors is essential for effective platelet adhesion to fibrin. On quiescent platelets, integrin αIIbβ3 is in its inactive conformation, in which the globular integrin head domain bent back toward the platelet cell membrane. Only upon platelet activation, such as by the precedent binding of vWF and collagen to their respective receptors, cytoplasmic adaptor molecules, particularly talin and kindlin, bind to the αIIbβ3 integrin (Moser et al., 2008; Kasirer-Friede et al., 2014). Via this so-called in-side out signaling, the unclasping of the cytoplasmic domains and membrane-proximal stalk domains of both integrin subunits as well as a conformational extension of the integrin ectodomains is induced. This goes along with an enhancement of binding affinity to the fibrin ligand (Arnaout et al., 2005; Luo et al., 2007; Huang et al., 2019; Humphries et al., 2019) and integrin clustering (Li et al., 2017), which eventually enables platelets to firmly attach to fibrin scaffolds and to exert contractile forces onto it (White, 2000; Haling et al., 2011).

## The Prothrombotic Microenvironment of Solid Tumors

Under healthy conditions, hemostasis aims to seal any damage of the otherwise leakproof circulatory system, to stop bleeding and to initiate tissue regeneration by providing a provisional ECM network of fibrin. These tasks are accomplished by the interdependent network of platelets, blood coagulation factors, and the blood vessel wall (Gale, 2011; Broos et al., 2012; Berndt et al., 2014).

**FIGURE 1:**
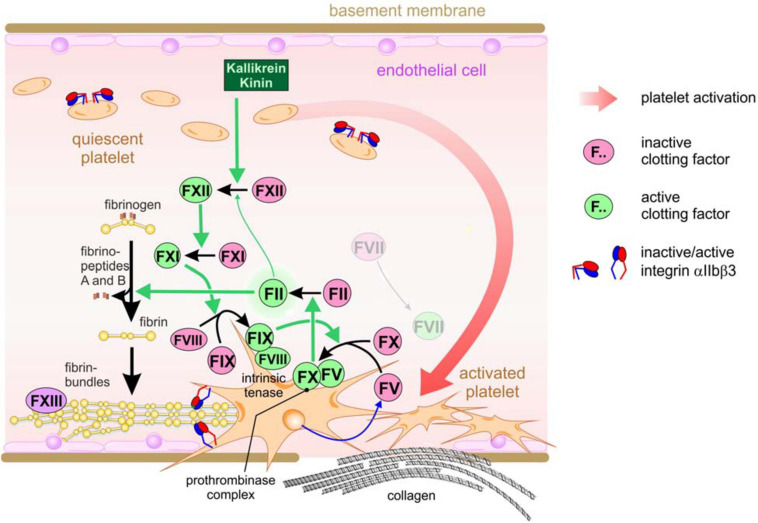
The intrinsic coagulation pathway resulting in the hemostatic sealing of a leaky blood vessel wall. Triggered by damaged vessel walls and by exposed negatively charged surfaces, kallikrein partially cleaves and thus activates coagulation factor XII, which subsequentially activates factor XI. As the next step in this activation cascade, factor XI activates factor IX, which together with activated factor VIII associates into the intrinsic tenase complex on the surface of activated platelets which have attached to the subendothelial basement membrane or collagen fibrils at the vessel injury site. The tenase complex activates factor X, which then forms another platelet-tethered complex together with platelet-secreted factor V, the protrombinase complex. This complex turns on thrombin (factor II), resulting in fibrinogen conversion and deposition of insoluble fibrin fibrils, which are further stabilized via covalent cross-linkage by factor XIII.

The final product of the coagulation pathway is fibrin (Jennewein et al., 2011; Swieringa et al., 2018; Kwaan and Lindholm, 2019). Coagulation comprises a complex network of serine proteinases, which float in the blood plasma in an inactive zymogenic form (Dahlbäck, 2000; Gale, 2011). Intrinsic hemostasis is triggered intravascularly by negatively charged surfaces and kallikrein, at injured sites of the vessel wall, where endothelial cells detach and leave a desnuded basement membrane (Figure 1). This triggers a cascade of partial proteolysis steps, by which clotting factors XII, XI, and IX activate each other sequentially (Gale, 2011). Platelets also attach to the denuded vessel wall, are activated and thus take on a dendritic cell shape. Activated factor IX associates with activated factor VIII in a Ca^2+^ ion-dependent manner on the surface of these activated platelets, a process which is supported by the enrichment of phosphatidylserine residues in the outer membrane leaflet (de Witt et al., 2014; Swieringa et al., 2018). This intrinsic tenase complex is named according to its substrate, clotting factor X (X being the Roman numeral for 10). It proteolytically cleaves factor X, which together with activated factor V forms yet another coagulant complex on the platelet surface, the prothrombinase complex, resulting in active thrombin (factor II) (Wojtukiewicz et al., 2016; Reddel et al., 2019). In addition to fibrinogen conversion, thrombin is able to activate several upstream clotting factors, thereby perpetuating and amplifying coagulation. Moreover, thrombin elicits intracellular signals by activating protease-activated receptors (PARs), thereby reinforcing platelet activation (Wojtukiewicz et al., 2015). In the context of hemostasis, it converts soluble fibrinogen monomers into insoluble fibrin molecules. They aggregate into highly ordered supramolecular bundles that form rope-like structures and networks, that stabilize the platelet aggregates and allow the platelets to contract the resulting thrombus (Gale, 2011). Factor XIII, a transglutaminase, secreted from the platelets, covalently crosslinks the fibrin molecules, thereby strengthening thrombus stability. Several control mechanisms are implemented into this network of coagulation to avoid spatially or temporarily inappropriate or overshooting hemostasis, which would cause vascular occlusion, thrombosis and consequentially ischemia (Connolly and Francis, 2013). Among them, thrombin triggers also the anticoagulant circuit of protein C activation to confine the coagulation cascade locally and temporally (Dahlbäck, 2000; Gale, 2011).

**FIGURE 2:**
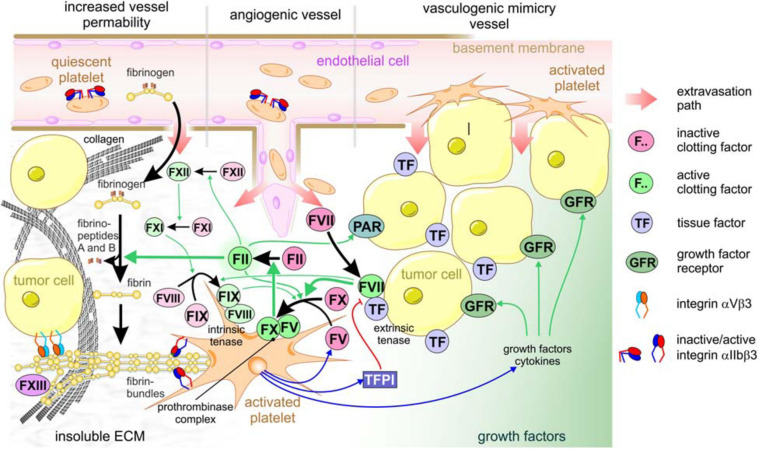
Platelets are crucial for tumor cell-induced coagulation and contribute to the TME of solid tumors. Increased vessel permeability in the vicinity of a tumor, tumor-induced angiogenic sprouting or vasculogenic mimicry vessels are three ways that allow extravasation of platelets and clotting factors from the blood stream into the interstitial stroma of tumor tissue. After the platelets recognize vWF of the subendothelial basement membrane, or collagen of the desmoplastic TME, platelets become activated, thereby take a dendritic shape, expose phosphatidylserine on the outer leaflet of cell membrane, degranulate, and activate the adhesion receptors, especially αIIbβ3 integrin. After leaking from the blood vessel, clotting factor VII immediately is activated and associates with tissue factor, which is abundant on most tumor cells, thereby forming the extrinsic tenase complex. This activates factor X and XI, the former of which forms the prothrombinase complex, together with platelet-secreted factor V, on the surface of activated platelets exposing phosphatidylserine. This activates thrombin (factor II). In addition to this main activation path, thrombin activation is perpetuated by integrating the intrinsic coagulation pathway: thrombin > factor XII > factor XI > intrinsic tenase complex (factor IX:FVIII) > factor X > thrombin. Also, PARs are activated by thrombin on tumor cells, additionally promoting tumor progression. Activated thrombin converts soluble fibrinogen from the blood plasma to insoluble fibrin molecules that aggregate into highly ordered fibrin bundles. They change the composition of the TME substantially and allow further platelets to adhere. Activated platelets secrete not only factor V and factor VIII, but also tissue factor pathway inhibitor (TFPI), thereby feeding back on the extrinsic tenase complex, as well as growth factors. Relevant growth factor receptors, e.g., for PDGF and TGFβ, provide proliferative signals to tumor cells.

The tight lining of endothelial cells prevents platelets and fibrinogen from getting in contact with extravascular protagonists that trigger activation of platelets and of coagulation factors (Nagy et al., 2012; Benazzi et al., 2014; Fitzgerald et al., 2018; Klein, 2018). During vessel damage and tissue injury, platelets and fibrinogen from the blood meet these prothrombotic and coagulant agents, thereby physiologically leading to hemostasis and to the generation of fibrin-scaffolded granulation tissue, which in normal tissue would be the first step in wound healing and tissue regeneration (Menter et al., 2017). However, where do platelets meet cancer cells that grow in a solid mass within epithelial or stromal tissue? How do platelets become part of the TME? The answers lay in the high permeability of blood vessels in the vicinity of a tumor mass (Nagy et al., 2012). This leakiness allows the intravascular platelets and fibrinogen to access the extravascular space, where platelets become activated and fibrinogen is converted to fibrin by coagulation (Figure 2). Depending on the type of tumor vessel, there are different ways, how tumor vessels lose their gatekeeper functions and allow extravasation of platelets and coagulation factors (Figure 2; Chouaib et al., 2010; Klein, 2018). First, the tumor mass approaches preexisting vessels due to its volume-demanding growth or by vessel co-option (Kuczynski et al., 2019; Niland and Eble, 2019). Vascular endothelial growth factor (VEGF), especially its isoform VEGF-A, and likely other chemokines from the TME increase the permeability of normal blood vessels and activate endothelial cells (Apte et al., 2019). This may result in increased leukocyte extravasion. Moreover, activation of endothelial cells is accompanied by the loss of antithrombotic surface molecules, such as thrombomodulin and heparin sulfate, from the endothelial cell surface (Klein, 2018). This increases the propensity of intravascular coagulation and thrombosis. Second, VEGF, produced by the cells of the tumor mass in response to the hypoxic TME, also stimulates angiogenesis. However, the ingrowing blood vessels are tortuous, variably calibered, and chaotically organized with a patchy and discontinuous lining of endothelial cells, while the underlying basement membrane is not fully established and defective, and the stabilizing mural cells, such as pericytes, are missing (Benazzi et al., 2014; Zanotelli and Reinhart-King, 2018). Thus, the irregular angiogenic vessels become another route for blood components to leak into the tumor mass (Figure 2). The blood flow in these vessels is irregular and likely turbulent, which also increases endothelium activation and leakiness. Vasculogenic mimicry (VM) vessels are a third option for extravasation of blood components into tumor tissue. Although their existence has been controversially discussed for the last two decades, VM vessels occur in a variety of solid tumors with a bad prognosis for the patient (Andonegui-Elguera et al., 2020; Wechman et al., 2020; Wei et al., 2021). These vessels are hardly or not at all lined by endothelial cells, but possess a sleeve of ECM glycoproteins. The latter are responsible for their detection with periodic acid fuchsin staining (PAS) reagent in histological sections. Moreover, in an attempt to confine the lumen of VM vessels, tumor cells align to shape a tube-like conduit. There, tumor cells are in contact with the blood flow and, due to their discontinuous cell-cell contacts, give another route of extravasation of blood components. Along these three routes, blood components reach the tumor mass and contribute with their high colloid-osmotic pressure to the characteristically elevated interstitial pressure within the TME. The rigidity and tension of the ECM adds to this high interstitial pressure typical of the TME (Mohammadi and Sahai, 2018; Eble and Niland, 2019).

The subendothelial basement membrane and the underlying three-dimensional ECM scaffold significantly contributes to the TME (Eble and Niland, 2019; Mongiat et al., 2019). In leaky blood vessels, it gives way for platelets and coagulation factors to reach the interstitial ECM scaffold of the tumor mass and the cancer cells (Acerbi et al., 2015; Naba et al., 2016, 2017). Within the TME, the coagulation cascade preferentially runs along the extrinsic pathway. In contrast to intrinsic hemostasis, it is alternatively triggered by tissue factor (TF) (factor III, thromboplastin, CD142), which is expressed on adventitial stroma cells and is abundant on tumor cells. It recruits factor VII, which leaks into the TME and, upon proteolytic activation, predominantly cleaves factor X, but also factor XI. Therefore, this complex on tumor cells is called the extrinsic tenase complex (Repetto and De Re, 2017; Chowdary, 2020). Activated factor X and platelet-released factor V form the prothrombinase complex on the platelet surface in a Ca^2+^ ion-dependent manner. This eventually activates thrombin (factor II), the central serine protease of coagulation (Figure 2).

Through the leaky tumor vessel, also platelets reach the tumor site in three subsequent steps: tethering, adhesion, and activation of platelets (Kunicki, 2001; Wong, 2013; Hansen et al., 2018; Scharf, 2018a). Platelets tether to subendothelial vWF, which is synthesized and secreted by endothelial cells. The shear forces of the turbulent blood flow in the tortuous and leaky tumor vessels change the conformation of vWF and enable its recognition by the GPIb-complex (Huck et al., 2014). This prepares the adhesion and activation steps of platelets, as tethering provides a sufficiently long period for other adhesion receptors to bind to additional ECM proteins. Then, due to their cytoskeleton rearrangement, platelets also actively migrate along the ECM into the TME (Wong, 2013). Cancer cells and their neighboring stroma cells change the composition and physical properties of the ECM in the tumor mass remarkably (Eble and Niland, 2019; Henke et al., 2019). Distinct ECM proteins are abundantly deposited in the TME, such as collagen fibrils, fibronectin variants EDA and EDB, and tenascin-W (Acerbi et al., 2015; Naba et al., 2016, 2017; Eble and Niland, 2019; Niland and Eble, 2020). They are deposited in gradients within the TME, with high concentrations of collagens and fibronectin in the periphery of the tumor mass, especially of desmoplastic tumors (Oudin and Weaver, 2016; Wang et al., 2016; Eble and Niland, 2019). Therefore, approaching the tumor from the leaky vasculature, platelets first experience and attach to collagen, either via vWF, which via its A3-domain decorates collagen, or directly via collagen receptors. Collagen belongs to the strongest activators of platelets. Activated platelets further migrate into the tumor mass, a process which is likely reinforced by the distribution of ECM proteins and by other gradients within the TME, such as mechanical stiffness and tension, or of metabolites, acidic pH and oxygen tension (Oudin and Weaver, 2016). Thus, platelets enter the TME and become a part of it.

Shortly after their discovery in 2004, neutrophil extracellular traps (NETs) were pinpointed as another way of platelet extravasation and infiltration into the TME (Cedervall et al., 2018; Masucci et al., 2020). Upon activation and arrest at a leaky tumor vessel, neutrophil granulocytes may undergo NETosis, a suicidal process, which results in the production of ROS and release of decondensed nuclear and mitochondrial DNA into the extracellular space. Histones are modified and released from the denuded chromosomal DNA leaving behind a network of nucleic acid, originally meant to entrap pathogens (Masucci et al., 2020). However, these highly negatively charged nucleic acids form the surface, on which clotting factor XII is activitated, and vWF-bound factor VIII is enriched. Activated factor XII initiates the intrinsic pathway of coagulation, also at the sites of leaky tumor vessels (Campello et al., 2018). Moreover, this nucleic acid network also entraps platelets and allows them to extravasate at these TME-near vessel sites (Erpenbeck and Schon, 2017; Cedervall et al., 2018). Although not entirely clear today, NET-mediated platelet entrapment is an alternative route of platelet recruitment to the TME.

## Cooperation of Tumor Cells and Platelets Within the Tme of a Solid Tumor Mass

Once in the physical vicinity of the tumor cells, platelets contribute to the TME in several ways (Gay and Felding-Habermann, 2011a; Menter et al., 2017). Activated platelets release clotting factors, V and VIII, which reinforce the coagulation cascade (Swieringa et al., 2018). In particular, by secreting factor V, platelets complement the activity of the extrinsic tenase complex on the cancer cells, as both activated factors V and X form the prothrombinase complex on platelets (Camire, 2016). By flipping phosphatidylserine to their outer leaflet of the cell membrane, platelets form the platform for thrombin generation. Thrombin takes multiple roles within the TME by activating other tumor secreted proteases, by activating PARs on both platelets and cancer cells (Wojtukiewicz et al., 2015), and by remodeling the ECM scaffold and turning it to a fibrin-rich matrix (Repetto and De Re, 2017; Kwaan and Lindholm, 2019). Moreover, platelets store multiple growth factors, such as PDFG, VEGF, and transforming growth factor-β (TGFβ), as well as several cytokines (Gay and Felding-Habermann, 2011a; Swieringa et al., 2018; Bikfalvi and Billottet, 2020). This cocktail of signaling molecules is secreted from activated platelets into the TME. VEGF supports angiogenesis of endothelial cells (Sabrkhany et al., 2011; De Palma et al., 2017; Apte et al., 2019). Certain platelet-derived cytokines suppress immune cells (Palacios-Acedo et al., 2019; Bikfalvi and Billottet, 2020). PDGF and TGFβ are strong stimulators for fibroblasts to differentiate into cancer-associated fibroblasts (CAFs) (Ostman, 2017; Lodyga and Hinz, 2020). CAFs convert the TME into a cancer cell-supportive environment by remodeling the ECM, by exerting mechanical forces and interstitial tension, and supporting tumor cell invasion (Kalluri, 2016; Eble and Niland, 2019; Pakshir et al., 2020). Another way of platelet-tumor cell-communications are microvesicles, which are secreted by platelets and are taken up by tumor cells in the TME (Goubran et al., 2015; Vajen et al., 2015).

A special cooperativity between platelets and tumor cells becomes evident in the formation of the extrinsic tenase complex on tumor cells. It contains tissue factor (TF), which is abundantly expressed on several tumor cells (Han et al., 2014; Versteeg, 2015; Grover and Mackman, 2018; Unruh and Horbinski, 2020). Moreover, its expression is upregulated in many tumor entities (Han et al., 2014 and references therein) and correlates with the malignancy grade of the tumors (Callander et al., 1992; Shoji et al., 1998; Liu et al., 2011; Zhang et al., 2020). Also, CAFs are a rich source of tissue factor and also form the extrinsic tenase complex, together with the factor VII that diffuses into the TME from the leaky blood vessels (Liu et al., 2011; Zhang et al., 2020). Although it is unclear whether this prometastatic effect of TF-FVII complex on tumor cells is caused by its proteolytic activity or its signaling function, practically, TF-inhibitory antibodies or FVII-deficiency reduce metastasis in murine breast cancer models (Versteeg, 2015; Rondon et al., 2019). Conspicuously, but not entirely understood is the regulatory interplay of tissue factor pathway inhibitor (TFPI), a Kunitz type domains-containing inhibitor of the tissue factor:factor VII-complex, which is secreted by activated platelets (Dahlback, 2017; Chowdary, 2020).

Two isoforms of tissue factor are generated by alternative splicing, a full length variant (flTF) and an alternatively spliced variant (asTF) (Unruh and Horbinski, 2020). They share the same N-terminal ectodomain, which encompasses two fibronectin type III-domains, each with an intradomain disulfide bridge. Whereas flTF is anchored in the cell membrane via a single span transmembrane domain, the asTF only peripherally tethers to the cell membrane via five positively charged amino acid residues at the shortened C-terminus. Three N-glycoconjugates may influence the association affinity for activated factor VII (Stone et al., 1995). The cytoplasmic domain of flTF may be phosphorylated and palmitoylated (Unruh and Horbinski, 2020). In addition to its coagulant function, the extrinsic tenase complex on cancer cells may also cleave ephrin receptors, ephB2 and ephA2 (Kania and Klein, 2016), or PAR2 (Wojtukiewicz et al., 2015). For instance, the latter initiates a signaling process of increased protein kinase C (PKC) activity and enhanced intracellular Ca^2+^ levels within the cancer cells (Unruh and Horbinski, 2020). Both flTF and asTF can also associate with distinct β1 integrins, thereby inducing a conformational change and consequential increase of ligand binding activity and cell migration (Versteeg, 2015).

Of remarkable impact on ECM remodeling within the TME is the ability of the extrinsic tenase complex to trigger the coagulative conversion of fibrinogen into fibrin. This is not restricted to the immediate surroundings of the cancer cells, as cancer cells intensely secrete microparticles which contain flTF and phosphatidylserine. These microparticles are abundantly found in the blood of cancer patients. Due to their strong procoagulant potential, their abundance correlates with the risk of venous thromboembolism (Tesselaar et al., 2009; Owens III, and Mackman, 2011) and may be a determinant in preparing premetastatic niches (Gkolfinopoulos et al., 2020).

At the primary tumor site, fibrin can be considered as a joint product of platelets and tumor cells, which changes the biochemical and biophysical composition of the TME (Eble and Niland, 2019). The fibrin network resembles the fibrin-rich ECM of granulation tissue during wound repair. Via cell adhesion receptors, it feeds back on both platelets and cancer cells (Kwaan and Lindholm, 2019). Whereas platelets attach to fibrin via their most numerous receptor, integrin αIIbβ3, tumor cells recognize fibrin via αVβ3 integrin (Katagiri et al., 1995). The binding sites for both integrin receptors are different from each other and located at different sites within the 50 nm long fibrin molecule, allowing both receptors to bind to the same molecule without steric competition (Springer et al., 2008). Especially for platelets, the interaction with fibrinogen and fibrin is a complex and regulated process, as both platelet and fibrinogen are blood plasma components without interacting with each other under normal conditions. Quiescent platelets usually bear αIIbβ3 integrin in an inactive, bent conformation (Xiao et al., 2004). It does not bind to fibrinogen nor to cleaved fibrin molecules in solution (Vickers, 1998). Instead, fibrin must form highly ordered fibrils and networks or must be immobilized to surfaces to allow effective binding of αIIbβ3 integrin and platelet adhesion (Podolnikova et al., 2010). This requirement is fulfilled only after coagulation within the TME. Therefore, network formation and deposition of fibrin changes the adhesive properties of the TME for both platelets and cells. It enables αIIbβ3 integrin to be conformationally activated (Xiao et al., 2004; Podolnikova et al., 2009; Durrant et al., 2017). Like other ECM receptors, fibrin-bound αIIbβ3 integrin molecules likely cluster, in line with the pattern of integrin binding sites within the fibrin network (Ozaki et al., 2013; Martins Lima et al., 2019). Via outside-in signaling, they convey the adhesive signal into the platelet by recruiting cytoskeletal, adaptor, and kinase proteins, such as Src and focal adhesion kinase (FAK), to the cellular attachment site (Coller, 2015; Durrant et al., 2017; Huang et al., 2019). Platelets, especially those that leak out of the blood stream after the first thrombotic events are activated and reinforce the thrombotic and coagulant TME. Such adhesive clues of the fibrin-enriched and remodeled ECM, together with the platelet-secreted growth factors, cytokines, and microvesicles decisively contribute to the TME and influence the TME-embedded cells, such as cancer cells, CAFs, immune cells and endothelial cells (Kalluri, 2016; Eble and Niland, 2019).

## Blood-Borne Tumor Cells Are Sheltered and Supported by Associating Platelets

**FIGURE 3:**
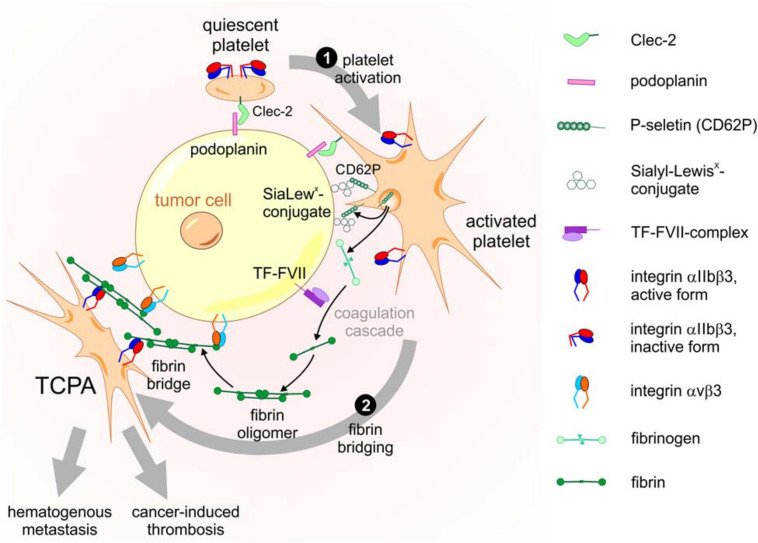
Molecular components and activation steps of the TCPA formation cascade. The cascade of TCPA formation consists at least of the following two steps. ❶ Platelet activation triggered by Clec-2-podoplanin interaction results in degranulation with surface exposure of P-selectin, release of fibrinogen and conversion of integrin αIIbβ3 into an active conformation. ❷ Fibrin bridging of platelets and tumor cells requires cleavage of fibrinogen to fibrin by the extrinsic coagulation pathway initiated by the TF-FVII complex on tumor cells. Fibrin molecules, aggregated in a network, are bound by αVβ3 and αIIbβ3 integrins on tumor cells and platelets, respectively, allowing a stable mechanical bridge within the TCPA.

The physical interaction of platelets with tumor cells is not only relevant in the TME of solid tumors, but also crucial in the formation of blood-borne tumor cell-platelet aggregates (TCPAs) and hematogenous metastasis. Immediately after entering the bloodstream, tumor cells are coated with platelets on their surface. This cloak protects them from the shear stress, that they experience from the blood flow (Egan et al., 2014), and prevents them from being eliminated by the immune system (Palumbo et al., 2007; Gay and Felding-Habermann, 2011b). The formation of TCPAs is mediated by receptors on platelets that bind to their counterreceptors on tumor cells and by the establishment of fibrin bridges between the two cell types (Erpenbeck and Schon, 2010; Schlesinger, 2018). In this process, several interactions are responsible for the firm cohesion of platelets to tumor cells, such as the binding between (i) C-type lectin-like receptor 2 (Clec-2) on platelets with podoplanin on tumor cells, (ii) P-selectin on activated platelets, and Sialyl-Lewis^x^-conjugates on tumor cells, and (iii) the integrins αIIbβ3 on platelets and αVβ3 on tumor cells, which both bind to a fibrin bridge. TCPA formation is a multistep cascade initiated by Clec-2 based platelet activation followed by fibrinogen conversion and fibrin bridging (Figure 3).

Clec-2 is an N-glycosylated type II membrane protein with a short cytoplasmic tail containing a hemi-immunoreceptor tyrosine-based activation motif (hemITAM) and a C-type lectin-like ectodomain (Nagae et al., 2014). It is exclusively expressed on the platelet membrane. Its counter piece is podoplanin, also known as aggrus, which appears in several tissues and is overexpressed on certain types of cancer cells (Suzuki-Inoue, 2019). Podoplanin is a type I membrane protein containing a short cytoplasmic tail which anchors the receptor to the actin cytoskeleton (Quintanilla et al., 2019) and an extracellular part harboring in tandem three copies of a platelet aggregation (PLAG) stimulating motif (Nagae et al., 2014). The third PLAG motif has an O-linked disialyl sugar moiety attached (Kaneko et al., 2007). Sialyation together with the acidic amino acids glutamate and aspartate in podoplanin‘s PLAG3 motif is essential for electrostatic contact with three arginine residues in Clec-2 and for stimulation of platelet aggregation, as revealed by structural analysis of the Clec-2-podoplanin protein complex (Nagae et al., 2014). Interaction with its agonist podoplanin results in translocation of Clec-2 into lipid rafts (Pollitt et al., 2010), mixtures of sphingolipids, cholesterol, and proteins that form membrane microdomains (Simons and Gerl, 2010). The dense packing in rafts mediates clustering of Clec-2 receptors (Ozaki et al., 2013). The multimerization of Clec-2 increases the affinity for podoplanin and in turn causes podoplanin clustering, resulting in a strong interaction between platelets and tumor cells (Pollitt et al., 2014).

Upon podoplanin-dependent Clec-2 clustering in lipid rafts, platelet activation is initiated by Clec-2 signaling through its hemITAM sequence (Suzuki-Inoue, 2019). The hemITAM segment consists of the tyrosine phosphorylation motif YXXL adjacent to an upstream triple of acidic amino acids (Hughes et al., 2013). Phosphorylation of the hemITAM YXXL is catalyzed by a collaboration between Syk tyrosine kinase and a Src family tyrosine kinase (Spalton et al., 2009; Severin et al., 2011; Pollitt et al., 2014). This enables Syk to avidly bind with its pair of Src homology (SH2) domains to the phosphorylated hemITAM sequences of two Clec-2 molecules that form a dimer (Watson et al., 2009). Thereby, the catalytic activity of the tyrosine kinase Syk is further boosted (Suzuki-Inoue, 2019). Eventually, Syk starts a downstream signaling cascade by phosphorylation of the adaptor proteins, LAT (linker for activation of T cells) and SLP-76 (Src homology 2 domain-containing leukocyte protein of 76 kDa), leading to activation of Btk (Bruton tyrosine kinase) and PLCγ2 (phospholipase C γ2). Finally, cleavage of phosphatidylinositol-4,5-biphosphate (PIP2) by PLCγ2 generates the second messenger molecules IP3 (inositol 1,4,5-triphosphate) and DAG (diacylglycerol), that increase intracellular Ca^2+^ levels and activation of PKC (protein kinase C), resulting in platelet activation (Pollitt et al., 2010; Suzuki-Inoue, 2019). Subsequently, additional intercellular interactions are established and further tighten the cohesion between platelets and tumor cells within TCPAs (Erpenbeck and Schon, 2010).

After initial Clec-2-podoplanin mediated activation, platelets degranulate and expose P-selectin on their cell membranes (Jenne et al., 2013; van Golen et al., 2015). P-selectin on the platelet surface binds to the Sialyl-Lewis^*x*^-tetrasaccharide conjugate of PSGL-1 (P-selectin glycoprotein ligand-1) expressed on tumor cells, thus constituting a secondary level of platelet-tumor cell cohesion downstream of the Clec-2-podoplanin interaction (Coupland and Parish, 2014).

Fibrin which is generated from fibrinogen by thrombin serves as another bracket between tumor cells and platelets as it is bound by integrin αIIbβ3 on the platelet surface and by integrin αVβ3 on the tumor cell, building a firm bridge between the two cell types (Katagiri et al., 1995). While integrin αVβ3 binds to an RGD motif in the fibrinogen α subunit, integrin αIIbβ3 interacts with another peptide sequence in the γ subunit (Springer et al., 2008). As a result, platelets and tumor cells firmly cohere to form stable TCPAs connected by three different receptor pairs: Clec-2 and podoplanin, P-selectin and PSGL-1, and integrin αIIbβ3 via fibrin to integrin αVβ3 (Figure 3). Moreover, clotting factor FXIII, a transglutaminase, crosslinks plasma fibronectin to insoluble fibrin oligomers, thereby forming a fibrin-fibronectin network (Corbett et al., 1997) and imbedding the metastasizing tumor cell. In addition, the fibrin-fibronectin complex binds to and further activates integrin αVβ3 on tumor cells, inducing invadopodia formation and metastasis (Malik et al., 2010). Yet another way of tumor cell-platelet crosstalk has been reported recently. CD97, an adhesion dependent G protein coupled receptor on tumor cells, activates platelets by a so far unknown counterreceptor to release lysophosphatidic acid (LPA) from dense granules (Ward et al., 2018). In turn, LPA binds to its receptor on tumor cells and starts the Rho kinase signaling pathway, which is associated with tumor cell invasiveness (Ward et al., 2018).

A major threat to blood-borne cancer cells during hematogenous metastasis are natural killer cells that clear tumor cells by cytolytic attack (Nieswandt et al., 1999). In the microenvironment of TCPAs, tumor cells exploit platelets to escape their elimination by the immune system (Labelle and Hynes, 2012; Schlesinger, 2018). The presence of MHC (major histocompatibility complex) class I molecules on the surface of a cell indicates “self” to avoid activation of the immune response. Since many tumor cells traveling alone in the blood circulation show no or low expression of MHC class I molecules on their surface, they are easy prey to natural killer cells (Placke et al., 2012b). In contrast, platelets express plenty of MHC class I molecules on their membrane, which shelters them and the close-by tumor cells within the TCPAs (Placke et al., 2012b). These platelet derived MHC class I molecules confer “self” to tumor cells and thereby circumvent destruction by natural killer cells (Placke et al., 2012a). Furthermore, activated platelets release growth factors such as PDGF and TGFβ from their α-granules. PDGF binds to PDGF receptors on the surface of natural killer cells and lowers their cytolytic activity (Gersuk et al., 1991). TGFβ impedes expression of NKG2D, a C-type lectin-like receptor on natural killer cells that normally detects transformed tumor cells (Kopp et al., 2009). Accordingly, this constricts cytotoxicity of natural killer cells and lowers disposal of interferon-γ, an anti-tumorigenic cytokine and stimulator of the immune response (Kopp et al., 2009).

## The Role of Platelets in Preparing Metastatic Niches

Having escaped the mechanical challenges in the blood circulation and the threat of the immune system, tumor cells in TCPAs receive further support by platelets as they move to their destination site. After floating for some minutes in the bloodstream, TCPAs come to rest at the vasculature of blood vessels, where tumor cells prepare for extravasation (Labelle and Hynes, 2012). To support attachment of their accompanying tumor cells to the vessel wall, activated platelets bind with P-selectins to Sialyl-Lewis^x^-conjugates on the surface of endothelial cells (Gay and Felding-Habermann, 2011a). Hence, platelets create a custom-made microenvironment to safeguard the tumor cells, help them in tethering to endothelial cells and expedite metastasis in their target tissue (Schlesinger, 2018; Palacios-Acedo et al., 2019; Gkolfinopoulos et al., 2020). Fostering the formation of metastatic niches, activated platelets release a multitude of growth factors, cytokines, ECM proteins and clotting factors, and attract different host derived cells of the myeloid lineage such as neutrophils, monocytes and macrophages to assist the piggy-backed tumor cells (Labelle and Hynes, 2012).

Platelets release cytokines CXCL5 and CXCL7 that bind to the CXCR2 receptor on neutrophils to attract them to the metastatic niche (Labelle et al., 2014; Wu et al., 2019; Jaillon et al., 2020). There, neutrophils bind to tumor cells and enforce their attachment to endothelial cells (Huh et al., 2010). Furthermore, upon stimulation by platelet delivered TGFβ, neutrophils produce reactive oxygen species (ROS), nitric oxide and arginase that prevent stimulation of immune reactive T cells (Jaillon et al., 2020). In addition, neutrophils release leukotrienes that confer a high tumorigenic and metastatic potential to tumor cells (Wculek and Malanchi, 2015). Moreover, tumor cells in TCPAs induce the secretion of acid sphingomyelinase from platelets, in turn leading to hydrolysis of sphingomyelin to ceramide (Carpinteiro et al., 2015). In the tumor cell, ceramide molecules form membrane domains in which receptor proteins, such as integrin α5β1, accumulate. Integrin α5β1 clustering further strengthens attachment of tumor cells to the endothelial wall and thereby promotes their metastatic potential (Carpinteiro et al., 2015).

TGFβ released from platelets and direct contact between platelets and tumor cells activate TGFβ-Smad and NF-κB pathways, respectively, in tumor cells (Labelle et al., 2011; Labelle and Hynes, 2012). This results in transcription of target genes, which promote transformation of tumor cells from an epithelial to a mesenchymal phenotype (Labelle et al., 2011; Mittal, 2018). Accordingly, synergistic activation of TGFβ-Smad and NF-κB pathways downregulate epithelial markers such as claudin1 and E-cadherin, but lead to upregulation of mesenchymal transcription factors such as snail and twist, and marker proteins including vimentin and fibronectin in tumor cells (Labelle et al., 2011; Mittal, 2018). Moreover, target genes of the NF-κB and TGFβ-Smad signaling pathways, such as Serpin family H member 1 (SERPINH1), Matrix metalloproteinase 9 (MMP9), Vascular endothelial growth factor C (VEGF-C), Tenascin C (TNC) and C-C motif chemokine 2 (CCL2) enhance hematogenous tumor metastasis by different mechanisms (Labelle et al., 2011; Kim et al., 2019; Xiong F. et al., 2020). For example, during epithelial-mesenchymal transition, snail dependent downregulation of E-cadherin results in loss of intercellular contacts between cancer cells, and loss of their original polarized cellular shape and tissue specific properties (Mittal, 2018). Therefore, tumor cells become mobile, allowing migration and extravasation through the endothelial barrier. Tumor cell overexpressed SERPINH1 encodes the endoplasmic reticulum resident heat shock protein 47 (Hsp47), a molecular chaperone that fosters collagen type I production and export through the secretory pathway during epithelial-mesenchymal transition (Xiong G. et al., 2020). This results in the collagen-rich desmoplastic TME also at the metastatic site. Accordingly, collagen enforces recruitment of platelets to tumor cells, thereby facilitating migration through the endothelium and induces interaction of tumor cells and cluster formation, a necessity for metastasis (Xiong F. et al., 2020). MMP9 is required for ECM remodeling, while VEGF-C and TNC are involved in endothelial cell growth and angiogenesis (Carmeliet, 2005; Rupp et al., 2016). CCL2 released from tumor cells after platelet stimulation is the ligand for chemokine receptor 2 (CCR2) expressed on monocytes and endothelial cells (Wolf et al., 2012). CCL2 binding attracts monocytes to tumor cells and confers suppression of the immune response (Mantovani and Sica, 2010). Stimulation of endothelial cells by CCL2 increases cell permeability of the endothelial wall and tumor cell extravasation by signaling via Janus kinase 2 (JAK2), which triggers downstream signal transducer and activator of transcription 5 (Stat5) and p38 mitogen-activated protein kinase (p38MAPK) activation (Wolf et al., 2012). Eventually, tumor cells that have acquired a mesenchymal, mobile phenotype break the endothelial barrier and make their way to form metastases at distant organs.

## Perspectives for Translation Into Clinical Treatment Strategies

Platelets and coagulation play a crucial role in tumor progression and hematogenous metastasis and are therefore considered as valid pharmaceutical targets in anti-cancer therapy (Zhang et al., 2020). The important surveillance of platelet counts during chemotherapy, their role in chemoresitance, and the potential use of platelets as drug delivery systems in chemotherapy have recently been reviewed (Swier and Versteeg, 2017; Dovizio et al., 2020; Zhang et al., 2020). Therefore, we will not focus on this platelet-dependent aspect of chemotherapy, but will here focus on targeting platelets as relevant constituents of the TME. With respect to the TME of solid tumor mass, different strategies have been chosen to target platelets and tumor cell-induced coagulation. Interestingly, a monoclonal antibody inhibiting TF-signaling via PAR2 reduces tumor growth, while another TF-directed antibody that inhibits its proteolytic function only impedes hematogenous metastasis (Versteeg et al., 2008). Another monoclonal antibody, CNTO 859, inhibits both progression and hematogenous metastasis of tumor cells (Ngo et al., 2007). Two recombinant anticoagulant proteins, the nematode anticoagulant protein c2 (NAPc2) and ixolaris from a tick species, both targeting the TF:FVII complex reduce also tumor progression and metastasis (Hembrough et al., 2003; Carneiro-Lobo et al., 2009). However, it is not entirely clear yet, which isoform of TF influences which steps of tumor progression and metastasis and via which mechanism (van den Berg et al., 2012). Also, TF-positive microvesicles are intensely studied as potential targets to reduce tumor-induced thrombosis and metastasis (Rondon et al., 2019).

Instead of inhibiting TF, another therapeutic approach utilizes TF to pharmaceutically induce tumor infarction. To this end, recombinant TF is directed to tumor vessels either in a soluble form or bound to nanoparticles (Schwöppe et al., 2010; Ding et al., 2013; Schliemann et al., 2020). By enriching TF in the tumor vessels, an excessive fibrin deposition and thrombus formation occlude the tumor vasculature (Jahanban-Esfahlan et al., 2017). The resulting hemostatic plug abolishes the blood flow and the nutrient support for the tumor, resulting in tumor infarction and regression of tumor tissue (Schliemann et al., 2020).

Targeting adhesion and infiltration of platelets within the TME of solid tumors is also tested as potential strategy to curb tumor progression. To this end, adhesive platelet receptors are molecular targets, especially as they have already been clinically addressed to treat thrombosis. RGD-mimicking compounds that interfere with ligand binding of both platelet integrin αIIbβ3 and αVβ3 on tumor cells are relevant lead structures for anti-tumor applications (Jamasbi et al., 2017; Miller et al., 2017). Their inhibitory mechanism and efficacy on RGD-dependent integrins have been derived from snake venom disintegrins (Huang et al., 2016; Estevao-Costa et al., 2018). Another toxin class from snake venoms, the C-type lectin-related proteins, are very efficient inhibitors specifically directed to the receptors for collagen and vWF (Eble, 2019).

The contact of quiescent platelets with blood-borne tumor cells initiates the formation of TCPAs, giving rise to hematogenous metastasis and cancer-induced thrombosis. Therefore, it is of upmost importance to interfere with this initial step of platelet activation right at the beginning (Figure 3). Since the primary contact between tumor cells and platelets likely is mediated by the Clec-2-podoplanin interaction, targeting this early step should substantially impede the entire TCPA formation cascade. Accordingly, several approaches are being followed to identify inhibitors of Clec-2 and podoplanin (Harbi et al., 2021). Among the Clec-2 targeting toxins, the snake venom rhodocytin, also named aggretin, was the first to be discovered (Suzuki-Inoue et al., 2006). It is a heterodimeric C-type lectin from the Malayan pit viper *Calloselasma rhodostoma* consisting of α and β chains covalently linked by a disulfide bridge. Crystal structure analysis identified rhodocytin as a (αβ)_2_ heterotetramer that binds to Clec-2, induces receptor clustering on the cell surface and thereby triggers platelet activation and aggregation (Watson et al., 2008). Mutations introduced into recombinant rhodocytin expressed in E. coli preserved heterodimer formation but disturbed the oligomeric suprastructure of rhodocytin (Sasaki et al., 2018). Remarkably, such rhodocytin mutant still binds to Clec-2 but fails to induce receptor clustering, thereby blocking the Clec-2-podoplanin axis and platelet activation (Sasaki et al., 2018). Furthermore, when tested in a mouse model of podoplanin-induced lung cancer, mutant rhodocytin prevented formation of metastases, demonstrating that change of its suprastructure can turn rhodocytin from an agonist to an antagonist of tumor cell-induced platelet aggregation and TCPA formation (Sasaki et al., 2018). Since protein medications are rather circumstantial to administer, small drug-like compounds are another approach that was followed to disrupt the Clec-2-podoplanin axis. Cobalt hematoporphyrin was identified by screening a chemical compound library for inhibitors of the interaction between recombinant expressed Clec-2 and podoplanin (Tsukiji et al., 2018). When cobalt hematoporphyrin was used in a mouse model, podoplanin-induced pulmonary metastasis was blocked efficiently (Tsukiji et al., 2018), however, at a potency yet too low for clinical application (Harbi et al., 2021). In a similar approach the 5-nitrobenzoate compound 2CP was discovered as another inhibitor of the Clec-2-podoplanin interaction (Chang et al., 2015). 2CP suppressed pulmonary tumor metastasis in a xenograft mouse model, although at concentrations yet too high for clinical use (Chang et al., 2015).

Another strategy to interfere with Clec-2-podoplanin mediated TCPA formation may be function-blocking antibodies. The mouse monoclonal antibody AYP1 specifically recognizes human Clec-2 and perturbs activation and aggregation of platelets upon stimulation with podoplanin or snake venom rhodocytin (Gitz et al., 2014). However, no *in vivo* study to test this antibody in cancer metastasis has been reported so far. Likewise, monoclonal antibodies 8.1.1 and SZ168 were raised against podoplanin that block the interaction with Clec-2 and platelet activation (Rayes et al., 2017; Wang et al., 2021). When used in a mouse model, podoplanin antibody SZ168 repressed podoplanin dependent, cancer associated thrombosis (Wang et al., 2021).

These preliminary data indicate that the TME provided by the recruitment of platelets to a solid tumor mass or to blood-borne cancer cells is a valid target to curb tumor progression and metastasis. Newly developed tools targeting the multiple platelet-tumor cell interactions may open new strategies not only to reduce the fatal consequence of the Trousseau syndrome but also to impede tumor growth and dissemination.

## Author Contributions

WO and JE drafted and wrote the manuscript. KB helped in search for references and edited the manuscript. All authors contributed to the article and approved the submitted version.

## Conflict of Interest

The authors declare that the research was conducted in the absence of any commercial or financial relationships that could be construed as a potential conflict of interest.
